# Does visuospatial neglect contribute to standing balance within the first 12 weeks post-stroke? *A prospective longitudinal cohort study*

**DOI:** 10.1186/s12883-023-03475-1

**Published:** 2024-01-22

**Authors:** Elissa Embrechts, Jonas Schröder, Tanja C. W. Nijboer, Charlotte van der Waal, Christophe Lafosse, Steven Truijen, Wim Saeys

**Affiliations:** 1 Department of Rehabilitation Sciences and Physiotherapy (REVAKI), Research Group MOVANT, University of Antwerp, Wilrijk, Belgium; 2 Department of Experimental Psychology, Helmholtz Institute, Utrecht University, Utrecht, The Netherlands; 3 Center of Excellence for Rehabilitation Medicine, UMC Brain Center, University Medical Center Utrecht, Utrecht University and De Hoogstraat Rehabilitation, Utrecht, The Netherlands; 4 Department of Neurorehabilitation, RevArte Rehabilitation Hospital, Edegem, Belgium

**Keywords:** Stroke, Visuospatial neglect, Longitudinal study, Posturography, Standing balance, Postural Control

## Abstract

**Background:**

Visuospatial neglect (VSN) has been suggested to limit standing balance improvement post-stroke. However, studies investigating this association longitudinally by means of repeated within-subject measurements early post-stroke are lacking. This prospective longitudinal cohort study evaluates the longitudinal association of egocentric and allocentric VSN severity with 1) standing balance independence and 2) postural control and weight-bearing asymmetry (WBA) during quiet standing, in the first 12 weeks post-stroke.

**Methods:**

Thirty-six hemiplegic individuals after a first-ever unilateral stroke were evaluated at weeks 3, 5, 8 and 12 post-stroke. Egocentric and allocentric VSN severity were evaluated using the Broken Hearts Test. The standing unperturbed item of the Berg Balance Scale (BBS-s) was used to clinically evaluate standing independence. Posturographic measures included measures of postural control (mediolateral (ML)/anteroposterior (AP) net center-of-pressure velocities (COPvel)) and WBA during quiet standing. A linear mixed model was used to examine longitudinal associations between egocentric and allocentric VSN, and BBS-s, COP_vel-ML_, COP_vel-AP_ and WBA within the first 12 weeks post-stroke.

**Results:**

Egocentric (β = -0.08, 95%CI[-0.15;-0.01], *P* = .029) and allocentric VSN severity (β = -0.09, 95%CI[-0.15; -0.04], *P* = .002) were significant independent factors for BBS-s scores in the first 12 weeks post-stroke. Egocentric and allocentric VSN were no significant independent factors for COP_vel-ML_, COP_vel-AP_ and WBA in the first 12 weeks post-stroke.

**Conclusions:**

Allocentric and egocentric VSN severity were significantly associated with decreased standing independence, but not impaired postural control or greater asymmetric weight-bearing, in the early subacute post-stroke phase. This may involve traditional VSN measures being not sensitive enough to detect fine-grained VSN deficits due to a ceiling effect between 5 and 8 weeks post-stroke, once the individual regains standing ability. Future studies may require more sensitive VSN measurements to detect such deficits.

Trial registration

Clinicaltrials.gov. unique identifier NCT05060458.

**Supplementary Information:**

The online version contains supplementary material available at 10.1186/s12883-023-03475-1.

## Introduction

Independent standing after stroke is an essential precursor to reacquiring walking ability [1, 2]. Post-stroke standing balance is characterized by underlying impairments in postural control such as increased postural sway of the center-of-pressure (COP) as compared to healthy controls, together with greater weight-bearing on the less-affected leg [3–7]. Apart from more severe impairments in lower-limb muscle strength [3], somatosensation [8], and age [3], cognitive deficits have been associated with deficient standing balance after stroke [9, 10]. Among these cognitive deficits, visuospatial neglect (VSN) stands out as a particularly striking condition.

VSN is characterized by a lateralized deficit in visuospatial cognition, awareness and attention, not attributable to sensorimotor or memory impairments [11]. VSN is common after stroke, with a reported prevalence ranging from 23 to 48% within the acute phase [12, 13]. Individuals with VSN typically exhibit reduced accuracy and larger latency to visual stimuli on one side of space, usually contralesional, as compared to the other [14]. The clinical presentation of the disorder is highly heterogeneous, such that VSN symptoms may manifest within different frames of references (egocentric/viewer-centered, allocentric/object-centered) and regions of space (personal/body, peripersonal/within-reach, extrapersonal/beyond-reach) [15]. Individuals with VSN after stroke tend to experience a slower recovery in activities of daily living and may have a reduced participation as compared to those without [16–18].

A recent systematic review highlighted that VSN has been associated to impaired sitting balance, as reflected by more dependency during sitting and a more asymmetric weight-bearing (weight-bearing asymmetry, WBA) when compared with individuals without VSN [9]. However, the association between VSN and standing balance using clinical and posturographic measures remains unclear. Some studies have shown that VSN was linked to impaired standing balance [19–25], whereas others did not [9, 20, 21, 26–28]. Additionally, only two studies have evaluated the association between VSN and standing balance longitudinally throughout the initial weeks post-stroke [29, 30], despite this being the period in which significant improvements in both VSN and balance are observed [10, 31]. Both studies merely evaluated the individual’s ability to perform the standing balance task on clinical scales (such as the Berg Balance Scale (BBS) [30] or a sit-to-stand task [29]), without providing insight into underlying postural control deficits or WBA. Consequently, the extent to which VSN contributes to underlying postural control and WBA during the early weeks post-stroke remains unknown.

The overarching aim of this study was to evaluate the longitudinal association of VSN with standing balance within the first 12 weeks post-stroke, by using a repeated measurement design with fixed assessment points relative to stroke onset. For this purpose, we applied posturographic measures of quiet standing by recording ground reaction forces (GRFs) and COP sway. To investigate the mechanisms underlying the possible association between VSN and standing balance, this study proposes the following research questions:

1. How is VSN severity associated with independence in terms of standing balance within the first 12 weeks post-stroke?
2. How is VSN severity associated with underlying postural control and WBA within the first 12 weeks post-stroke?

Regarding our first question, we hypothesized that VSN would be longitudinally associated with decreased standing balance, such that individuals with more severe VSN would also exhibit decreased independence in standing. Regarding our second question, we expected that VSN severity would also be associated with greater deficits in underlying postural control, as reflected by increased COP sway and greater weight-bearing on the less-affected leg. Additionally, we hypothesized that for questions 1 and 2, the proposed longitudinal associations would be independent, such that VSN would remain a significant contributor to standing balance independence, postural control, and WBA after controlling for several covariates, including lower limb strength [3], presence of sensory loss [8], and age [3].

## Methods

### Study design

This longitudinal prospective cohort study is part of a larger research project, entitled TARGET (Temporal Analyses of hemiplegic Gait and standing balance Early post sTroke; for protocol see) [32]. The protocol is registered online (ClinicalTrials.gov identified: NCT05060458), and the study was conducted in conformity with the STROBE statement.

### Subjects

Between October 2019 and December 2021, individuals admitted to one of the cooperating hospitals and rehabilitation facilities (Algemeen Ziekenhuis Geel, GZA Sint-Augustinus, GZA Sint-Vincentius, Universitair Ziekenhuis Antwerpen, RevArte) in the larger Antwerp region, Belgium, for acute or rehabilitation care after an ischemic or hemorrhagic stroke were screened for participation. Potential candidates were included when adhering to the following criteria: 1) CT/MRI-confirmed first-ever unilateral hemispheric stroke with onset less than 3 weeks ago, 2) Reduced muscle strength in the most affected lower limb, defined as a Motricity Index lower extremity score (MI-LE) of < 91 (i.e., at least “movement against resistance but weaker” in one item) at inclusion, 3) Pre-morbid independence in basic activities of daily life (i.e., modified Rankin Scale < / = 1), 4) Aged between 18 and 90 years old, 5) No severe orthopedic condition of the lower limbs and trunk or other neurological illness, 6) No severe cognitive or communication deficit that interferes with understanding of instructions, and 7) (Corrected to) normal visual acuity. These criteria are similar to those of the TARGET project for maintaining sample consistency and comparability [32, 33]. Screening and recruitment were performed by EE and JS together with the (para)medical staff employed at the stroke units and rehabilitation facilities.

### Procedures

All procedures were conducted in accordance with the Declaration of Helsinki and were approved by the Medical Ethics Committee of the University Hospital Antwerp (No. 18/25/305; Belgium trial registration no. B300201837010). Additional approval was obtained from the medical ethics committee of other involved sites. After receiving information, all subjects provided written informed consent for participation.

### Measurement procedures

Serial measurements were scheduled for each subject at week 3, 5, 8 and 12 post-stroke. At inclusion, subjects’ sex, age, stroke side (left/right) and type (ischemic/hemorrhagic) were recorded. At each time-point, VSN measurements, clinical measurements and, once independent standing was achieved, posturographic evaluations were performed. Also the clinical covariates (lower limb strength and sensory loss) were evaluated at each timepoint. Two trained assessors (EE and JS) administered clinical measures (including clinical covariates), and all serial measurements of an individual subject were conducted by the initial assessor. VSN measurements were performed by EE and posturographic measurements by JS.

#### VSN measurements

We evaluated both egocentric and allocentric VSN. Egocentric VSN is defined as the impaired ability to report visual stimuli on the neglected, usually contralesional, side of space. Allocentric VSN is defined as the difficulty in perceiving object features on the neglected side regardless of the object’s spatial position [13]. We used the Broken Hearts Test or its variation (Apple’s test) for VSN assessment, which is part of the Oxford Cognitive Screen [34]. Three parallel versions were used and varied randomly across time points to avoid learning effects. This test is recommended for VSN screening and screens for both subtypes [34–36]. It is a paper-and-pencil task in which the individual must cancel complete hearts/apples (n = 50) among distractors shaped as broken hearts/apples with either gaps on the right (*n* = 50) or left (*n* = 50) of the contour. It is presented on an A4 landscape paper, whose position is standardized within and across subjects. The paper was attached on a table, centrally and in front of the seated subject. The task was always performed with the less-affected hand, [34, 36] and subjects had a maximum of 3 min to complete the task.

#### Clinical measurement of standing balance

The activity scale for balance evaluation included the “standing unsupported” item of the Berg Balance Scale (BBS-s; score 0–4) [37]. The BBS-s evaluates standing independence, by asking the individual to stand without use of an aid or physical support for 2 min, without falling or requiring stepping responses due to instability. Higher scores indicate better performance [37].

#### Posturographic measurements

Postural control and WBA were assessed by instructing subjects to stand as still as possible for 40 s while keeping the arms alongside the trunk and eyes fixed at a non-moving visual target placed centrally in front of the subject. The bare feet were always positioned with 8.4 cm heel-to-heel distance and 9 degrees toe-out angle. No further instruction was given regarding weight-bearing symmetry. The first 10 s were removed from each trial to avoid starting effects and, if tolerated, at least three trials were performed with resting breaks in-between. To record ground reaction forces and COP excursions, we either used two floor-mounted force plates (Type OR6-7 Biomechanics Force Platform, AMTI, MA, US) at the *M*^*2*^*OCEAN* movement analyses laboratory (University of Antwerp, BE), or a portable plantar pressure plate (0.5 m Footscan pressure plate 3D, RS Scan, Materialize, BE). The latter allowed data collection in clinical environments when access to our laboratory was restricted. Prior to the current study, we performed a comparability study of the two measurement instruments in healthy controls during vision-deprived stance. This yielded strong consistency by Pearson correlation, yet systematic differences, in line with previous studies [38, 39]. Therefore, repeated measurements *within* a specific subject were always performed using the same instrument type, and statistical analyses of pooled data *between* subjects were corrected using INSTRUMENT as a covariate (see statistical analyses). COP excursions were computed using custom-written Matlab scripts (force plate data) or the system’s own software (pressure plate data). COP signals were subsequently low-pass filtered with a 10Hz second-order Butterworth filter [40].

### Outcome variables

#### Dependent variables

The dependent variable to evaluate research question 1 was the BBS-s (score 0–4), a measure of standing independence. For research question 2, dependent variables were measures of postural control and WBA. To quantify postural control, we calculated the root mean square COP velocity in mediolateral and anteroposterior sway directions (COP_vel-ML_, COP_vel-AP_; in mm/s) [41]. This measure was shown to be reliable and valid, by being sensitive to higher-frequent changes in the COP signal reflecting the process of posture stabilization [42]. In addition, WBA (%) was calculated by dividing the average vertical GRF below the more-affected leg by half of the total GRF under both feet combined. A percentage score of 0 indicates perfect symmetry and a positive or negative score reflect, respectively, a greater load on the less- or most-affected leg. All outcomes were averaged to improve reliability [40].

#### Independent variables

##### VSN outcome variables

The difference between cancelled full outlines on the ipsilesional vs. contralesional side of the paper was used as a measure of egocentric VSN severity and hereafter referred to as *egocentric asymmetry*. Egocentric VSN was considered present when egocentric asymmetry was > 2 or < -2. Allocentric VSN severity was calculated by subtracting the number of contralesional and ipsilesional gap false positives, which is referred to as *allocentric asymmetry*. Allocentric VSN was considered present when allocentric asymmetry was > 1 or < -1. Positive values indicate contralesional VSN and negative values indicate ipsilesional VSN [13, 34].

##### Clinical covariates

Lower limb strength was evaluated using the Motricity Index of the Lower-Extremity (MI-LE) [43]. The MI-LE (0–100) was measured by asking subjects to produce a maximum voluntary torque in the direction of hip flexion, knee extension, and ankle dorsiflexion. It is a valid and reliable scale [43]. Sensory impairment at the contralesional foot was assessed by applying light pressure touch at 6 points of the contralesional foot, using the Erasmus MC revised Nottingham Sensory Assessment protocol [44]. Sensory impairment was considered present when at least 2 points on the contralesional foot were missed.

### Statistical analyses

Statistical analyses were performed for subjects for whom data from at least two measurement points were available. We descriptively presented mean values with standard deviation of demographic information and each investigated outcome measure (i.e., egocentric asymmetry, allocentric asymmetry, BBS-s, MI-LE, sensory loss, COP_vel-ML_, COP_vel-AP_, WBA) at week 3, 5, 8, and 12 post-stroke.

#### Longitudinal association of VSN severity with clinical or posturographic measures

To investigate longitudinal associations between VSN severity and either clinical (BBS-s) or posturographic measures (i.e., COP_vel-ML_, COP_vel-AP_, and WBA), we fitted linear mixed models with the same model architecture for each dependent variable. The covariate TIME (categorical, four levels: weeks 3, 5, 8, and 12) was added as a fixed effect. A subject-specific random intercept was included to account for the dependency between the repeated within-subject measurements. Egocentric asymmetry or allocentric asymmetry were entered as independent variables in separate models. Before adding egocentric asymmetry or allocentric asymmetry as independent variables, we calculated the Spearman correlation coefficients between both factors to account for multicollinearity. This showed that both VSN subtypes were independent subtypes (*r* = 0.09, *P* = 0.297), justifying separate models. For posturographic measures, we accounted for systematic differences in COP between measurement instruments by adding an additional covariate, INSTRUMENT. The obtained regression coefficients (β) show the change in, respectively, BBS-s, COP_vel-ML_, COP_vel-AP_ or WBA by a one-unit increase in either egocentric asymmetry or allocentric asymmetry, respectively. The analysis technique of the linear mixed model allows the inclusion of patients with partial data missing at random [45].

#### Hierarchical model analyses

We further assessed whether the contribution of egocentric or allocentric asymmetry to the outcome variables remained significant after incorporating other relevant covariates, including MI-LE, SENSORY LOSS (yes/no), and AGE, by using a hierarchical linear mixed model. This was carried out solely for outcome measures that exhibited significant longitudinal associations with egocentric and/or allocentric asymmetry in the prior analysis. The proportional change in the β-estimates of egocentric asymmetry and allocentric asymmetry to the outcome after adding subsequent covariates (order: MI-LE, SENSORY LOSS (yes/no), AGE) was evaluated. To evaluate whether this would result in a better model fit, we evaluated change in model statistics using the sample size adjusted Akaike Information Criterion (AICc), with lower values indicating better fit.

#### Assessing potential ascertainment bias and its impact on standing independence

To control for potential ascertainment bias, which refers to the possibility that some subjects are more likely to be included in posturographic analysis than others because of their clinical status, we plotted the time courses of egocentric asymmetry and allocentric asymmetry for subjects who were and were not able to perform posturographic measurements (i.e., obtained a score of 4 on the BBS-s). To minimize the potential for bias in analysis, we re-ran the model for the BBS-s in subjects with available posturographic measures. For those without available posturographic measures, the statistical power was too low to yield meaningful results, and these were consequently excluded from further analyses.

All analyses were performed using JMP Pro® version 16. Histograms and Q-Q plots of residuals were inspected to confirm model assumptions.

## Results

### Subjects

**Fig. 1:**
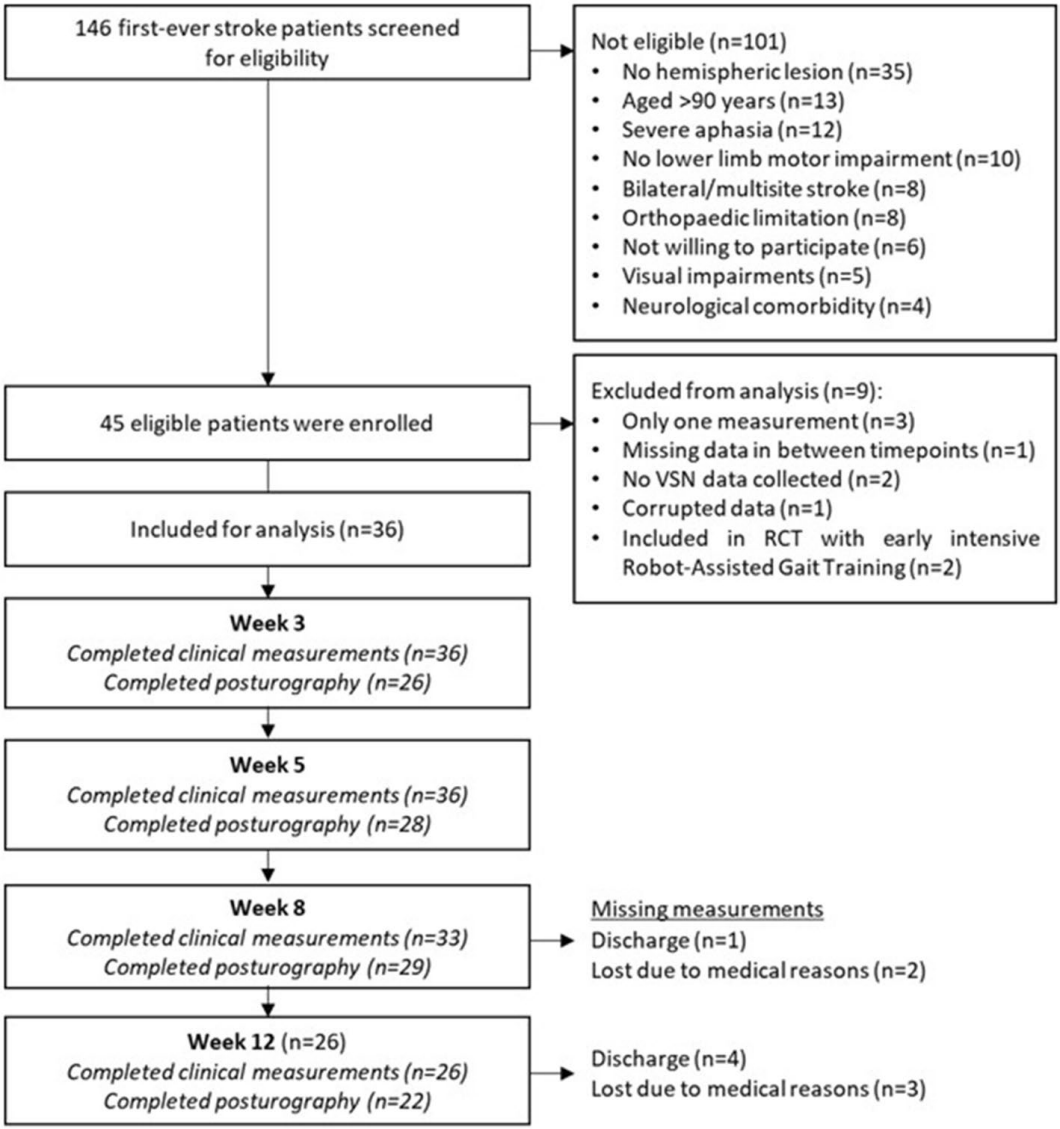
Flowchart of screening, inclusion and follow-up

Figure 1 shows the flow of subject recruitment. Approximately 180 first-ever stroke survivors were identified as potential candidates, of whom 45 adhered to the inclusion criteria and were successfully included. Of these, 36 successfully participated in at least two subsequent measurements and were included in the statistical analyses. Table 1 shows their baseline characteristics and Table 2 shows the mean values of each outcome variable at weeks 3, 5, 8, and 12 post-stroke. The mean age of the 36 included subjects was 59.78 (SD 15.96); 17 were female, 22 had a left-sided stroke, and 28 had an ischemic stroke. As shown, 14 individuals showed egocentric VSN at week 3, 7 at week 5, 9 at week 7 and 3 at week 12. Four individuals showed allocentric VSN at week 3, 6 at week 5, 3 at week 8, 4 at week 12.

**Table 1:** Subject characteristics at 3 weeks post-stroke

	Total
Age (years)	59.78 (15.96)
Sex (female/male)	17/19
Body weight, kg	75.39 (14.06)
Lesion side (left/right)	14/22
Stroke type (ischemic/hemorrhagic)	28/8
Time post-stroke (days)	24.56 (1.93)

Values are mean (standard deviation)

**Table 2:** Characteristics of subjects at 3, 5, 8 and 12 weeks

	**Week 3**	**Week 5**	**Week 8**	**Week 12**
Time post-stroke (days)	24.56 (1.93)	38.74 (2.12)	59.06 (2.31)	88.0 (4.42)
Egocentric asymmetry (0–20)°	2.97 (3.92)	2.03 (4.04)	1.85 (2.51)	1.08 (1.06)
Number of subjects with/without egocentric VSN	14/12	7/29	9/24	3/23
Allocentric asymmetry (0–20)°	1.83 (5.02)	0.56 (1.13)	0.42 (1.03)	0.73 (1.12)
Number of subjects with/without allocentric VSN	4/32	6/30	3/30	4/22
BBS-s score (0–4)	2.44 (1.75)	2.89 (1.51)	3.39 (1.06)	3.73 (0.53)
MI-LE (0–100)	57.42 (22.39)	65.00 (21.14)	71.09 (21.91)	72.31 (19.52)
Sensory loss (yes/no/NM)	9/18/9	8/19/9	6/20/7	4/16/6
N	26	28	29	10
Measurement instrument (FP/PP)	6/20	6/22	5/24	4/18
COP_vel-ML_ (mm/s)	7.62 (7.51)	6.31 (6.00)	5.72 (5.58)	5.01 (4.48)
COP_vel-AP_ (mm/s)	8.92 (7.45)	8.48 (8.00)	7.59 (6.76)	7.24 (6.36)
WBA (%)	43.65 (7.31)	44.40 (8.05)	43.04 (7.89)	43.75 (6.59)

*BBS-s* Berg Balance Scale – standing item, *COP*_vel-AP_ COP velocities in anteroposterior direction, *COP*_vel-ML_ COP velocities in mediolateral direction, *FP* Force plate, *MI-LE* Lower extremity part of the Motricity Index, *N* Number, *NM* Not mentioned, *PP* Pressure plate, *SD* Standard deviation, *VSN* Visuospatial neglect, *WBA* Weight-bearing asymmetry. Absolute values i.e., values irrespective of contra or ipsilesional side, otherwise these would cancel each other out. Values are mean (standard deviation)

### Longitudinal association of VSN with clinical measures of standing balance independence

As shown in Table 3, egocentric asymmetry (β = -0.11; [-0.17;0.06], *P* < 0.001) and allocentric asymmetry (β = -0.10; 95%CI[-0.16; 0.03]; *P* = 0.002) were significant factors for BBS-s within the first 12 weeks post-stroke.

**Table 3:** Linear mixed models for activity measures and postural control parameters

**Model with egocentric asymmetry as independent variable**						
**Dependent variables**		**Independent variables**				**AICc**
	**Egocentric asymmetry** **(β -value (SE, 95%CI,** ***p*-value))**	**Time (β-value (SE, 95%CI,** ***p*-value))**			**Instrument**	
		**3w**	**5w**	**8w**	**(β-value (SE, 95%CI,** ***p*-value))**	
**BBS-s**	**-0.11** (0.03, [-0.17;-0.06], *P* < .001)*	**-1.11** (0.23, [-1.57;-0.67], *P* < .001)*	**-0.78** (0.21, [-1.21;-0.35], *P* < .001)*	-0.24 (0.22, [-0.67;0.20], *P* = .280)		385.85
**COP**_**vel-ML**_	-0.29 (0.18, [-0.65;0.08], *P* = .119)	**3.37** (0.78, [1.81;4.92], *P* < .001)*	1.42 (0.75, [-0.06;2.91], *P* = .061)	0.84 (0.73, [-0.62;2.30], *P* = .255)	**9.29** (2.06, [5.07;13.51], *P* < .001)*	576.16
**COP**_**vel-AP**_	**-0.41** (0.17, [-0.75;-0.07], *P* = .018)*	**2.37** (0.72, [0.93;3.81], *P* = .002)*	1.17 (0.69, [-0.21;2.55], *P* = .095)	0.44 (0.68, [-0.92;1.79], *P* = .523)	**12.32** (2.33, [7.57;17.08], *P* < .001)*	572.34
**WBA**	-0.14 (0.26, [-0.66;0.38], *P* = .585)	-0.68 (1.12, [-2.91;1.55], *P* = .545)	1.02 (1.07, [-1.11;3.15], *P* = .344)	0.06 (1.05, [-2.03;2.15], *P* = .953)	-3.79 (3.22, [-10.37;2.80], *P* = .249)	656.96
**Model with allocentric asymmetry as independent variable**						
**Dependent variables**		**Independent variables**				**AICc**
	**Allocentric asymmetry** **β -value (SE, 95%CI,** ***p*-value)**	**Time (β-value (SE, 95%CI,** ***p*-value))**			**Instrument**	
		**3w**	**5w**	**8w**	**(β-value (SE, 95%CI,** ***p*-value))**	
**BBS-s**	**-0.11** (0.03, [-0.17;-0.06], *P* < .001)*	**-1.11** (0.23, [-1.57;-0.67], *P* < .001)*	**-0.78** (0.21, [-1.21;-0.35], *P* < .001)*	-0.24 (0.22, [-0.67;0.20], *P* = .280)		385.85
**COP**_**vel-ML**_	0.24 (0.30, [-0.34;0.83], *P* = .413)	**3.08** (0.77, [1.55;4.61], *P* < .001)*	1.33 (0.76, [-0.19;2.85], *P* = .085)	0.54 (0.72, [-0.89;1.97], *P* = .456)*	**9.64** (2.04, [5.48;13.80], *P* < .001)*	578.00
**COP**_**vel-AP**_	0.30 (0.28, [-0.26;0.86], *P* = .290)	**1.95** (0.73, [0.50;3.40], *P* = .009)*	1.01 (0.72, [-0.42;2.45], *P* = .163)	-0.00 (0.68, [-1.35;1.35], *P* = .998)	**12.80** (2.28, [8.13;17.46], *P* < .001)*	577.04
**WBA**	-0.29 (0.41, [-1.11;0.54], *P* = .487)	-0.94 (1.07, [-3.08;1.20], *P* = .382)	0.78 (1.06, [-1.34;2.90], *P* = .464)	-0.12 (1.00, [-2.12;1.87], *P* = .902)	3.83 (3.25, [-10.47;2.81], *P* = .248)	656.76

Each row represents the respective model for a certain dependent variable. *AICc* Sample size adjusted Akaike Information Criterion, *BBS-s* Berg Balance Scale – standing unsupported item, *CI* Confidence interval, *COP*_vel-AP_ anteroposterior center-of-pressure velocities, *COP*_vel-ML_ Mediolateral center-of-pressure velocities, *SE* Standard error, *W* Weeks, *WBA* Weight-bearing asymmetry, *β* Estimate, *P < .05

### Longitudinal association of VSN with posturographic outcomes of standing balance

Table 3 shows that egocentric asymmetry was a significant factor for COP_vel-AP_ (β = -0.41, 95%CI[-0.75; 0.07], *P* = 0.018), but not for COP_vel-ML_ (β = -0.29, [-0.65; 0.08], *P* = 0.119) and WBA (β = -0.14, [-0.66; 0.38],* P* = 0.585). Allocentric asymmetry was not a significant factor for COP_vel-ML_ (β = 0.24, [-0.34;0.83], *P* = 0.413), COP_vel-AP_ (β = 0.30, 95%CI [-0.26; 0.86], *P* = 0.290) and WBA (β = -0.29, [-1.11; 0.54], *P* = 0.487).

### Hierarchical model to evaluate influence of covariates on longitudinal associations and prediction errors

Table 4 shows that egocentric asymmetry (β = -0.08, 95%CI[-0.15;-0.01], *P* = 0.029) and allocentric asymmetry (β = -0.09, 95% CI[-0.15; -0.04], *P* = 0.002) maintained significant after adding MI-LE, SENSORY LOSS, and AGE to the BBS-s scores throughout the first 12 weeks post-stroke. The addition of these covariates resulted in a proportional change of -27.27% in the β-estimate of egocentric asymmetry and -10.00% in the β-estimate of allocentric asymmetry and decreased the estimated prediction error by 28.13% and 30.83%, respectively, for the prediction of BBS-s. In contrast, egocentric asymmetry did not remain a significant factor for COP_vel-AP_ after adding the MI-LE.

**Table 4:** Hierarchical model with addition of Motricity Index, sensory loss and age

	**Hierarchical models with egocentric asymmetry included as an independent variable**						
**Dependent variable**	**Model**	**Independent variable**		**Covariates**			**AICc (change%)**
		**β Egocentric asymmetry** **(SE, 95%CI,** ***p*-value)**	**β Egocentric asymmetry change**	**β MI-LE** **(SE, 95%CI,** ***p*-value)**	**β Sensory loss** **(SE, 95%CI,** ***p*-value)**	**β Age** **(SE, 95%CI,** ***p*-value)**	
BBS-s	Standard	**-0.11** (0.03, [-0.17;-0.06], *P* < .001)*					385.85
	Model 1	**-0.11** (0.02, [-0.16;-0.07], *P* < .001)*	0%	**0.04** (0.01, [0.03;0.05], *P* < .001)*			352.38 (-8.67%)
	Model 2	**-0.08** (0.04, [-0.15;-0.01], *P* = .027)*	-27.27%	**0.03** (0.01, [0.02;0.05], *P* < .001)*	**0.57** (0.32, [-0.07;1.20], *P* = .079)*		275.00 (-28.73%)
	Model 3	**-0.08** (0.04, [-0.15;-0.01], *P* = .029)*	-27.27%	**0.03** (0.01, [0.02;0.04], *P* < .001)*	**0.58** (0.32, [-0.07;1.22], *P* = .078)*	-0.01 (0.00, [-0.03; 0.02], *P* = .617)	277.30 (-28.13%)
COP_vel-AP_	Standard	**-0.41** (0.17, [-0.75;0.07], *P* = .018)*					572.34
	Model 1	-0.29 (0.17, [-0.63;0.06], *P* = .106)	-29.27%	**-0.12** (0.03,[-0.18;-0.05], *P* < .001)*			563.02 (-1.63%)
	Model 2	0.04 (0.15, [-0.26;0.34, *P* = .815)	-109.76%	**-0.07** (0.03; [-0.12;-0.01], *P* = .021]*	**-3.34** (1.00, [-5.33;-1.34], *P* = .001)*		409.72 (-28.41%)
	Model 3	0.04 (0.15, [-0.26;0.34, *P* = .793)	-109.76%	**-0.07** (0.03; [-0.13;-0.01], *P* = .017)*	**-3.37** (1.01, [-5.37;-1.37], *P* = .001)*	-0.05 (0.06, [-0.16;0.07], *P* = .396)	411.57 (-28.09%)
	**Hierarchical models with allocentric asymmetry included as an independent variable**						
**Dependent variable**	**Model**	**Independent variable**		**Covariates**			**AICc (change%)**
		**β Allocentric asymmetry** **(SE, 95%CI, p-value)**	**β Allocentric asymmetry change**	**β MI-LE** **(SE, 95%CI, p-value)**	**β Sensory loss** **(SE, 95%CI, p-value)**	**β Age** **(SE, 95%CI, p-value)**	
BBS-s	Standard	**-0.10** (0.03; [-0.16;-0.03]; *P* = .002)*					392.58
	Model 1	**-0.07** (0.03, [-0.13;-0.02], *P* = .008)*	-30.00%	**0.04** (0.01, [0.02;0.05], *P* < .001)*			365.03 (-7.02%)
	Model 2	**-0.09** (0.03, [-0.15;-0.04], *P* = .001)*	-10.00%	**0.03** (0.01, [0.01;0.04], *P* < .001)*	**0.79** (0.31, [0.17;1.41], *P* = .013)*		269.10 (-31.45%)
	Model 3	**-0.09** (0.03, [-0.15;-0.04], *P* = .002)*	-10.00%	**0.03** (0.01, [0.01;0.04], *P* < .001)*	**0.79** (0.31, [0.17;1.41], *P* = .013)*	-0.00 (0.01, [-0.02;0.02], *P* = .724)	271.54 (-30.83%)

For each dependent variable, hierarchical models are presented, starting with the standard model (no covariates and only VSN severity as independent variable) and ending with Model 3 (also including MI-LE, sensory loss and age as covariates). *Abbreviations*: *AICc* Sample size adjusted Akaike Information Criterion, *BBS-s* Berg Balance Scale – standing item, *COPvel-AP* Anteroposterior center-of-pressure velocities, *Model 1* Model with VSN and motricity index scores, *MI-LE* Lower extremity part of the Motricity Index, *Model 2* Model with VSN, Motricity index and sensory loss, *Model 3* Model with VSN, motricity index, sensory loss and age, *SE* Standard error, *β* Estimate

### Assessing potential ascertainment bias and its impact on standing independence

**Fig. 2:**
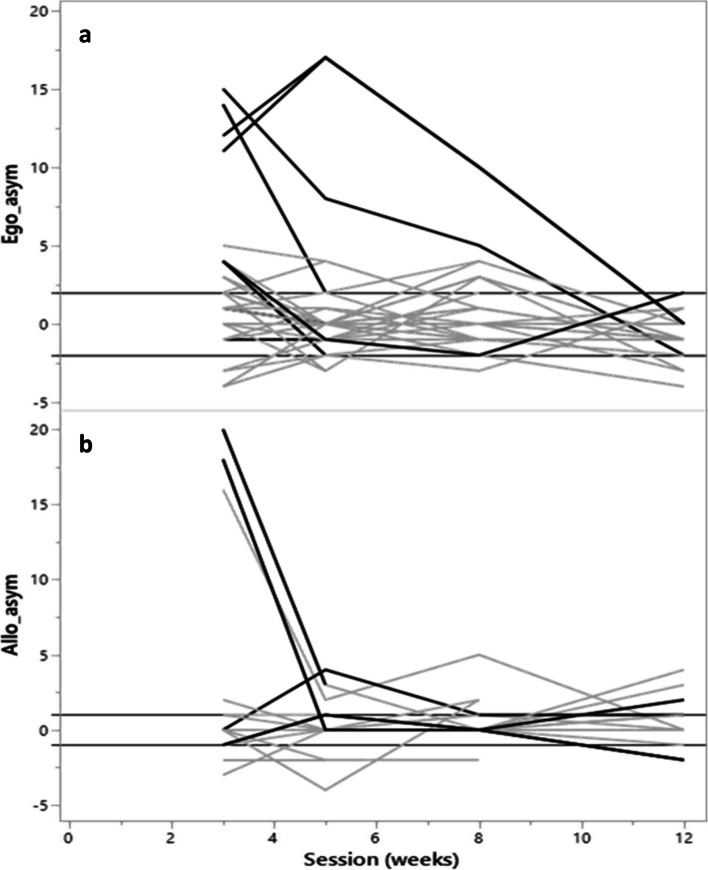
**a**-**b** Time course of egocentric asymmetry and allocentric asymmetry in subjects that were or were not able to perform posturographic measures. Abbreviations: Allo_asym: allocentric asymmetry, Ego_asym: egocentric asymmetry. Light grey lines show the time courses of egocentric asymmetry and allocentric asymmetry in those who were able to perform posturographic measures. Dark grey lines show the time courses for those who were unable to perform posturographic measures

Figure 2 shows that three subjects in the egocentric asymmetry and four subjects in the allocentric asymmetry graph were unable to undergo posturographic measurements. It is essential to consider the potential influence of ascertainment bias on our evaluation of standing independence.

To minimize the potential for bias in analysis, we re-ran the model for the BBS-s in subjects with available posturographic measures. This analysis demonstrated that for those subjects, neither egocentric asymmetry (β = 0.00, 95% CI [-0.089; 0.08], P = 0.916) nor allocentric asymmetry (β = -0.06, 95% CI [-0.19; 0.07], *P* = 0.375) emerged as significant predictors of the BBS-s.

## Discussion

The present prospective cohort study evaluated the association of egocentric and allocentric VSN with 1) standing balance independence and 2) postural control and WBA during quiet standing, within the first 12 weeks post-stroke. Our main findings were that:

- Both egocentric and allocentric asymmetry were significant independent factors longitudinally associated with decreased standing independence in the first 12 weeks post-stroke.
- Egocentric and allocentric asymmetry severity did not significantly contribute to impaired postural control or WBA in the first 12 weeks post-stroke.
- When correcting for potential ascertainment bias, egocentric and allocentric asymmetry were no longer significantly associated with standing independence.

VSN remained a significant and independent predictor of decreased standing independence, even after controlling for various covariates, which confirms our first hypothesis. The finding is congruent with those from a prior prospective cohort study conducted by Van Nes and colleagues [30], which also demonstrated that egocentric VSN severity was accompanied with reduced standing and walking independence in the first 3–6 months post-stroke. However, our study extends this one by controlling for important covariates including the strength of the most-affected leg, sensory loss, and age in a multivariate way. Moreover, to the best of our knowledge, this is the first study to examine the relative contribution of both egocentric and allocentric VSN on standing balance recovery post-stroke, as previous studies have only focused on the association between egocentric VSN and standing balance after stroke [9]. Our findings suggest that both aspects contribute to poor standing balance independence.

Despite this finding, a lack of an independent longitudinal association with underlying impaired postural control and WBA was found. This is opposing our second hypothesis, and suggests that once a subject resumed independent standing, VSN did not independently contribute to deficits in postural control, as reflected by exaggerated COP sway. This may further indicate that delayed achievement of independent standing in individuals exhibiting VSN would result from factors other than impaired postural control. Furthermore, VSN did not independently contribute to WBA within the first 12 weeks post-stroke. This indicates that an asymmetric stance with greater loading of the less-affected leg is not an expression of reduced attention to the most-affected side and a consequent shift in the representation of the mid-sagittal plane toward the less-affected side, as suggested previously [21]. Instead, it may merely reflect a compensatory strategy that favors the stronger, less-affected leg for balance control, due to reduced muscle strength in the most-affected leg [33].

Alternatively, the absence of a longitudinal association of VSN with postural control deficits and WBA may result from the observation that subjects with more severe (initial) VSN were unable to participate in posturographic measures, especially at the 3-week timepoint, as subjects must have the ability to stand independently to conduct such analyses**.** Potentially, once subjects with initial moderate-to-severe VSN (here measured using the Broken Hearts Test) reach standing ability, VSN may no longer be detectable on such tests. We observed that the VSN severity scores reached a ceiling effect between 5 and 8 weeks. Residual finer-grained impairments in lateralized visuospatial attention beyond this time window could not be demonstrated [46–48], making it difficult to establish significant associations between VSN recovery and underlying postural control deficits and WBA. This highlights that investigating how fine-grained changes in VSN beyond this time window contribute to postural control deficits and WBA over time poses a significant challenge for this field of research.

### Limitations

Several limitations of this study should be acknowledged. One limitation is the small sample size together with the dropout rate from 8 weeks onwards (27.8%), which was due to medical reasons and difficulties in scheduling measurements in the clinical setting after early discharge. In addition, COVID-19 measures prohibited the subjects’ outpatient access to the clinical sites. The limited sample size may have led to an underpowered analysis, potentially affecting our ability to establish statistical significance. Furthermore, only few subjects showed large deviations in VSN which may have further limited our results. Despite these limitations, our study's significant findings remain robust, and the *p*-values of the non-significant results consistently remained well above the significance threshold (α = 0.05). Nevertheless, our study underscores the need for future research with more substantial sample sizes. A second limitation is that the assessments of subjects were not initiated until 3 weeks post-stroke which may have resulted in missing early changes in the association of VSN with standing balance. Furthermore, posturographic measurements started even beyond this time-point in the more severely-affected subjects, which may have limited our findings. Third, posturographic data were collected using two different instruments. We believe that this did not affect our findings, considering we used the same instrument within subjects and added an extra covariate INSTRUMENT within the final analyses. Fourth, our study was constrained by the unavailability of more comprehensive lesion information, including details on etiology, severity, and topography. This limitation restricted our capacity to thoroughly assess the impact of lesion characteristics on the observed associations. Fifth, the linear mixed model approach used in the present study is that it combines within-subject and between-subject associations, which may limit the ability to fully understand the mechanisms driving the observed longitudinal associations between VSN severity and standing balance independence. Lastly, the BBS-s is a widely used tool, but it is a categorical measure rather than a continuous one, which may limit its sensitivity.

### Clinical implications and suggestions for further research

The results of this study show that both egocentric and allocentric VSN contribute to standing balance independence within the first 12 weeks post-stroke. Given that independent standing is a prerequisite for walking, it emphasizes the clinical importance of conducting a comprehensive assessment of both subtypes of VSN. Notably, VSN is more severe in the early weeks after a stroke, highlighting the critical need for early and targeted detection [31]. Beyond the first 5 weeks post-stroke, it becomes crucial to incorporate more sensitive measures for VSN detection, which may involve tasks demanding heightened attention, as demonstrated by Bonato and colleagues [49]. These tasks could load more intensively on individuals' attentional resources, complicating the deployment of compensatory strategies [50].

The present study could not explain *how* visuospatial neglect was longitudinally associated with standing balance independence within the first 12 weeks post-stroke. Being able to stand independently is a multifactorial skill, and our study shows that by multiple factors contribute to it throughout the first weeks post-stroke, including lateralized visuospatial attention, lower limb muscle strength and sensory function at the most-affected side. However, it is important to note that additional factors could have contributed to the observed longitudinal association between visuospatial neglect and standing balance independence. This could include factors such as multisensory integration [51], visual dependency [52], or an impaired perception of verticality [22, 53, 54]. While previous studies have suggested links between VSN, balance, and verticality misperception [22, 53, 54], no comprehensive investigation has assessed this relationship using standardized posturographic assessments over time. Future studies should evaluate whether these factors would explain the significant association between VSN and standing independence over time.

Secondly, our study was only able to evaluate associations, and was unable to determine causality. Therefore, future studies should investigate whether targeted interventions designed to improved VSN symptoms would lead to improved standing balance independence over time. These studies should also consider exploring the impact of lesion characteristics on this association, which would provide valuable insights into the benefits of interventions aimed at addressing VSN within rehabilitation. Barrett and colleagues [55] have already suggested to evaluate the effectiveness of this approach, especially given the suggested suppressive impact of VSN on upper limb motor recovery [56].

## Conclusion

Severity of egocentric and allocentric VSN was longitudinally associated with decreased standing independence in the first 12 weeks post-stroke. However, no significant longitudinal associations with postural control and WBA during quiet standing were observed. This suggests that the mechanisms underlying poor standing independence in individuals with VSN should involve other factors. However, this finding may have been influenced by the observation that the subjects with initially more severe VSN were unable to perform posturographic measurements. Consequently, evaluating postural control and WBA in those with initial moderate-to-severe VSN poses a significant challenge. Given that VSN may not be detectable anymore on classical paper-and-pen tests once these individuals regain standing ability, future research on standing balance recovery should implement more sensitive VSN measures that can detect residual impairments beyond this time window.

### Supplementary Information

**Additional file 1. **Supplementary table of demographic, clinical and posturographic data per subject.

## Data Availability

Data is available upon reasonable request.
