# Supplementary material for: Does visuospatial neglect contribute to standing balance within the first 12 weeks post-stroke? A prospective longitudinal cohort study
Source: BMC Neurol. 2024 Jan 22;24:37. doi: 10.1186/s12883-023-03475-1 (PMC10801963; doi:10.1186/s12883-023-03475-1)
Supplement: Supplementary file 1 — Additional file 1. Supplementary table of demographic, clinical and posturographic data per subject. [file 12883_2023_3475_MOESM1_ESM.docx]

**Supplementary table of demographic, clinical and posturographic data per subject**

| **ID** | **Age** | **Biological sex** | **Lesion side** | **Type** | **Ego asym** | **Allo asym** | **MI total** | **MI hip** | **MI knee** | **MI ankle** | **Sensory loss** | **BBS-s** | **TCT-s** | **FAC** | **RMI** | **Posturography avail?** | **COPvel-ML (mm/s)** | **COPvel-AP (mm/s)** | **WBA perc** |
| --- | --- | --- | --- | --- | --- | --- | --- | --- | --- | --- | --- | --- | --- | --- | --- | --- | --- | --- | --- |
| **1** | 61 | M | L | i | -4 | 0 | 64 | 25 | 25 | 14 | NT | 4 | 2 | 3 | 8 | 1 | 16.03 | 26.51 | 46.21 |
| **2** | 76 | F | L | i | 0 | 0 | 75 | 25 | 25 | 25 | NT | 4 | 2 | 5 | 10 | 1 | 5.14 | 11.35 | 42.63 |
| **3** | 78 | F | R | i | 12 | -1 | 58 | 25 | 19 | 14 | NT | 0 | 2 | 0 | 3 | 0 |  |  |  |
| **4** | 73 | F | R | i | 2 | -2 | 42 | 14 | 14 | 14 | NT | 3 | 2 | 0 | 3 | 1 | 19.39 | 18.44 | 33.24 |
| **5** | 49 | M | R | i | 0 | 0 | 83 | 25 | 33 | 25 | NT | 4 | 2 | 5 | 14 | 1 | 8.95 | 16.05 | 43.78 |
| **6** | 82 | F | R | i | 4 | 0 | 42 | 14 | 14 | 14 | NT | 1 | 2 | 1 | 4 | 1 | 22.38 | 20.20 | 16.42 |
| **7** | 40 | F | L | h | -1 | 0 | 32 | 9 | 14 | 9 | NT | 0 | 2 | 0 | 2 | 0 |  |  |  |
| **8** | 78 | M | R | i | -4 | 0 | 47 | 14 | 19 | 14 | NT | 2 | 2 | 2 | 6 | 1 | 28.47 | 28.58 | 40.90 |
| **9** | 68 | M | R | i | 3 | 0 | 99 | 33 | 33 | 33 | No | 4 | 2 | 5 | 14 | 1 | 1.51 | 4.13 | 48.60 |
| **10** | 77 | F | R | i | 2 | 16 | 53 | 14 | 14 | 25 | No | 0 | 2 | 1 | 3 | 0 |  |  |  |
| **11** | 42 | F | L | i | -1 | -1 | 61 | 14 | 33 | 14 | No | 4 | 2 | 3 | 9 | 1 | 2.22 | 3.86 | 43.35 |
| **12** | 74 | M | L | h | 3 | -3 | 75 | 25 | 25 | 25 | Yes | 3 | 2 | 2 | 5 | 1 | 4.43 | 11.37 | 51.35 |
| **13** | 46 | F | R | i | 15 | 0 | 42 | 14 | 14 | 14 | Yes | 0 | 2 | 0 | 1 | 0 |  |  |  |
| **14** | 56 | M | L | i | 2 | 0 | 52 | 19 | 19 | 14 | No | 4 | 2 | 4 | 9 | 1 | 5.42 | 5.22 | 43.41 |
| **15** | 46 | M | L | h | 2 | 0 | 52 | 14 | 19 | 19 | No | 3 | 2 | 2 | 6 | 1 | 10.33 | 9.38 | 36.39 |
| **16** | 20 | F | R | i | 0 | 1 | 91 | 25 | 33 | 33 | No | 4 | 2 | 5 | 14 | 1 | 1.60 | 1.91 | 48.20 |
| **17** | 69 | M | L | i | -1 | 2 | 63 | 19 | 25 | 19 | No | 3 | 2 | 3 | 6 | 1 | 3.63 | 3.84 | 51.30 |
| **18** | 60 | M | R | i | 1 | 0 | 47 | 14 | 14 | 19 | Yes | 2 | 2 | 1 | 6 | 1 | 11.91 | 7.27 | 42.26 |
| **19** | 65 | M | R | i | 14 | 0 | 18 | 9 | 9 | 0 | Yes | 0 | 2 | 0 | 2 | 0 |  |  |  |
| **20** | 59 | M | R | i | 0 | 0 | 47 | 14 | 14 | 19 | Yes | 4 | 2 | 4 | 8 | 1 | 5.41 | 6.60 | 38.98 |
| **21** | 67 | F | R | i | 4 | 20 | 0 | 0 | 0 | 0 | Yes | 0 | 2 | 0 | 1 | 0 |  |  |  |
| **22** | 40 | M | L | h | 0 | 1 | 9 | 0 | 9 | 0 | Yes | 0 | 2 | 0 | 3 | 0 |  |  |  |
| **23** | 69 | F | R | i | 5 | 0 | 83 | 33 | 25 | 25 | No | 4 | 2 | 3 | 6 | 1 | 2.87 | 5.75 | 49.02 |
| **24** | 36 | F | L | h | 2 | 0 | 75 | 25 | 25 | 25 | No | 4 | 2 | 5 | 13 | 1 | 1,63 | 2,81 | 46.63 |
| **25** | 51 | M | R | i | 0 | 0 | 59 | 25 | 25 | 9 | No | 4 | 2 | 4 | 7 | 1 | 5,79 | 6,72 | 46,39 |
| **26** | 82 | F | R | i | 0 | 0 | 83 | 33 | 25 | 25 | No | 4 | 2 | 4 | 9 | 1 | 1,57 | 3,47 | 49,70 |
| **27** | 24 | F | R | h | 0 | 0 | 83 | 25 | 33 | 25 | No | 4 | 2 | 4 | 11 | 1 | 3,34 | 3,09 | 45,63 |
| **28** | 66 | M | L | h | 1 | 0 | 52 | 19 | 19 | 14 | Yes | 0 | 2 | 0 | 2 | 0 |  |  |  |
| **29** | 75 | F | R | h | 1 | 0 | 63 | 19 | 19 | 25 | No | 1 | 2 | 1 | 5 | 1 | 3,98 | 4,48 | 39,23 |
| **30** | 46 | M | R | i | -3 | 0 | 58 | 14 | 25 | 19 | Yes | 2 | 2 | 1 | 5 | 1 | 19,97 | 14,01 | 52,73 |
| **31** | 60 | M | L | i | -3 | 0 | 69 | 25 | 25 | 19 | No | 4 | 2 | 4 | 13 | 1 | 2,03 | 2,95 | 40,45 |
| **32** | 65 | M | L | i | 0 | 0 | 83 | 25 | 33 | 25 | No | 4 | 2 | 4 | 7 | 1 | 5,54 | 7,33 | 49,13 |
| **33** | 52 | M | L | i | 1 | 0 | 63 | 19 | 25 | 19 | No | 4 | 2 | 2 | 6 | 1 | 2,55 | 3,60 | 43,62 |
| **34** | 57 | F | R | i | 1 | 0 | 63 | 19 | 19 | 25 | No | 4 | 2 | 4 | 8 | 1 | 2,14 | 2,92 | 45,31 |
| **35** | 66 | M | R | i | 4 | 18 | 23 | 9 | 14 | 0 | No | 0 | 2 | 0 | 1 | 0 |  |  |  |
| **36** | 77 | F | R | i | 11 | -1 | 58 | 25 | 19 | 14 | NT | 0 | 2 | 0 | 2 | 0 |  |  |  |
| Abbreviations: ego asym: egocentric asymmetry, allo asym: allocentric asymmetry, MI total: total score on Motricity Index (lower limbs), MI hip/knee/ankle: Motricity Index – hip/knee/ankle subscore, BBS-s: Berg Balance Scale – Standing item, TCT-s: Trunk Control Test-static, FAC: Functional Ambulation Categories, RMI: Rivermead Mobility Index, COPvel-ML/AP: Net center-of-pressure velocities in mediolateral/anteroposterior direction, WBA perc: percentage of weight-bearing asymmetry | | | | | | | | | | | | | | | | | | | |
